# Addressing intra-tumoral heterogeneity and therapy resistance

**DOI:** 10.18632/oncotarget.11875

**Published:** 2016-09-06

**Authors:** Brad Rybinski, Kyuson Yun

**Affiliations:** ^1^ Albert Einstein College of Medicine, Bronx, NY, USA; ^2^ The Jackson Laboratory, Bar Harbor, ME, USA; ^3^ Present Address: Houston Methodist Research Institute, Department of Neurosurgery, Houston, TX, USA

**Keywords:** epigenetic, heterogeneity, therapy resistance, cancer stem cells, microenvironment

## Abstract

In the last several years, our appreciation of intra-tumoral heterogeneity has greatly increased due to accumulating evidence for the co-existence of genetically and epigenetically divergent cancer cells residing in different microenvironments within a tumor. Herein, we review recent literature discussing intra-tumoral heterogeneity in the context of therapy resistance mechanisms at the genetic, epigenetic and microenvironmental levels. We illustrate the influence of tumor microenvironment on therapy resistance and epigenetic states of cancer cells by highlighting the role of cancer stem cells in therapy resistance. We also summarize different strategies that have been employed to address various resistance mechanisms at genetic, epigenetic, and microenvironmental levels in preclinical and clinical studies. We propose that future personalized cancer therapy design needs to incorporate dynamic and comprehensive analyses of tumor heterogeneity landscape and multi-dimensional mechanisms of therapy resistance.

## INTRODUCTION

With the advent of cost- and time-efficient genomic technologies, it may be possible to significantly improve patient outcomes by individualizing treatment regimes based on the genomic profiles of each patient tumor, the goal of precision medicine. However, this premise is based on two fundamental assumptions. One, that appropriate targeted therapies to all potential driver mutations/pathways will be available in the clinic. Two, that it will be possible to accurately and comprehensively profile each patient tumor to identify all critical driver mutations. Although much progress has been achieved in these areas, we have yet to fulfill these requirements. First, the currently available arsenal of FDA-approved targeted therapies falls short of covering the entire spectrum of already known oncogenic drivers, not to mention yet to be discovered oncogenic drivers. Much further research and drug development will be required before a more comprehensive collection of therapies becomes available. Second, recent studies have demonstrated significant intra-tumoral heterogeneity, i.e., co-existence of different genetically and epigenetically distinct malignant cells within the same patient tumor. Intra-tumoral heterogeneity has profound clinical implications and challenges current methods of tumor diagnosis and treatment. Because several excellent reviews presenting the evidence for intra-tumoral heterogeneity have been published recently [1, 2], this review will primarily focus on how different types of tumor heterogeneity contribute to therapy response and summarize strategies that have been used to address them. We have organized our discussion of tumor heterogeneity at three conceptual levels—genetic, epigenetic, and microenvironmental—although it is clear that these are intimately interconnected processes that significantly influence one another.

**Figure 1:**
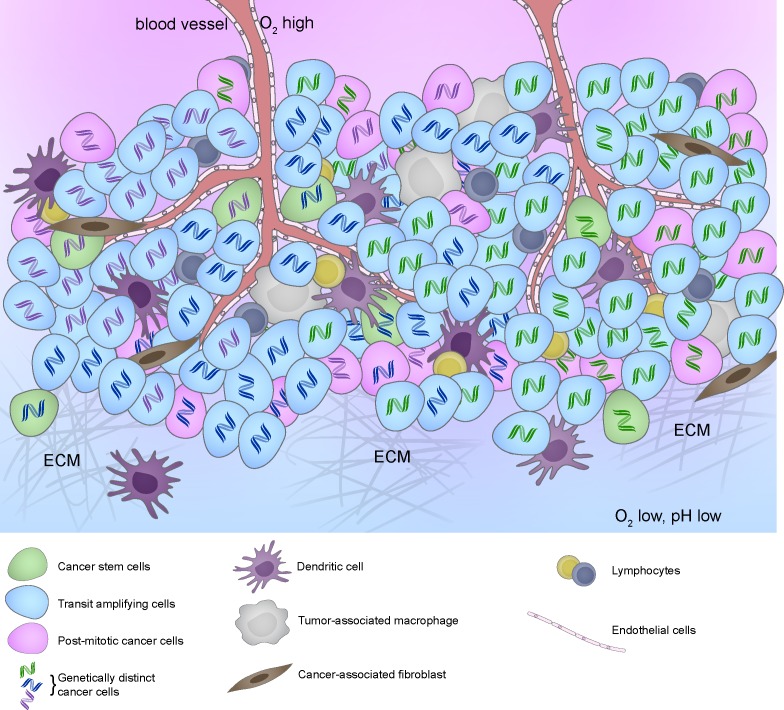
Intratumoral heterogeneity represented by cancer cells with different DNA color (genetic) and different cytoplasm color (epigenetic) in the context of different tumor microenvironment resulting from different stromal cell compositions and biophysical properties such as differences in extracellular matrix composition (stiffness), perfusion (hypoxia and acidosis), and other factors

The goal of personalized medicine is to match patients to therapies that are specific to oncogenic drivers in their tumors, resulting in treatments that are potentially less toxic and more effective. However, while many patients show an impressive initial response to targeted therapies, some patients are unresponsive despite having targeted mutation or pathway activation and others acquire resistance to treatment over time and relapses. A major culprit of therapy resistance appears to be intra-tumoral heterogeneity, arising from pre-existence of therapy-insensitive clones or therapy-induced mutations [1, 3]. Adding another layer of complexity, a heterogeneous local microenvironment further augments intra-tumoral heterogeneity by influencing the phenotypes of various cancer and non-cancer cells [1, 3–5](Figure 1). This extensive intercellular variation, or intra-tumoral heterogeneity, provides many opportunities for different mechanisms of drug resistance to emerge (Table 1).

**Table 1:** Resistance mechanisms and sensitization strategies

Genetic Heterogeneity	Resistance Mechanism	Sensitization or Elimination Strategy
	Mutations that prevent drug-target binding	Second or third generation inhibitors that bind at different sites or have increased affinity for the drug target [14, 24]
	Mutations upstream or downstream of the target molecule	Target multiple nodes in the same pathway [26, 28]
	Mutations that activate compensatory pathways	Inhibit multiple parallel targets [135]
	Mutations that affect P53 and its regulators	Degradation of mutant p53 [136] MDM2 inhibitors or inhibitors of MDM2-p53 interaction [137] p53 based immunotherapy [137] Targeting pathways downstream of mutant p53 [137]
**Epigenetic Heterogeneity**	**Resistance Mechanism**	**Sensitization or Elimination Strategy**
	Cancer stem cell phenotype	Aldehyde dehydrogenase inhibition [138] Cell cycle induction [139] Differentiation therapy [139]
	Core resistance phenotype common to CSC and EMT	HDACis and DMTis [86, 87] Hedgehog, Notch, TGF-β, Wnt-β catenin inhibition [66, 139] Metformin [139, 140] STAT-3 inhibition [141, 142]
	EMT phenotype	Abl/Srcinhibitor dasatinib [143] AXL inhibition [144] Targeting EMT associated transcription factors [66]
	Survival pathway activation and evasion of cell death	Inhibition of pro-survival proteins such as BCL-2 family members [145], heat shock proteins [146], and Survivin [147] PARP-1 and other DNA repair protein inhibitors [148] P-glycoprotein and drug efflux pump inhibitors[149]
**Microenvironment Heterogeneity**	**Resistance Mechanism**	**Sensitization or Elimination Strategy**
	Abnormal vasculature resulting in impaired delivery of systemic therapy, immunological effectors, and oxygenated blood	Anti-angiogenic therapy for tumor vessel normalization [100] Chloroquine [150] Dopamine [151] Pharmacological restoration of endothelial cell junctions [152]
	Dense extracellular matrix resulting in impaired delivery of systemic therapy	Extracellular matrix normalization by angiotensin II receptor blockers [100] Hyaluronan depletion [153] Hyaluronan and liposomal drug formulations [153, 154]
	Exosomes	Pharmacological inhibition of exosome release [129]
	Hypoxia	Many strategies are being explored; see [103] for review
	Soluble RTK Ligands (Resistance to targeted therapy)	Co-targeting of multiple kinases [128] Chemotherapy drugs such as cisplatin [128]
	Survival signaling induced by ECM attachment	BCL-2 inhibition [111] FAK and Src inhibition [155, 156] Integrin targeting [157]
	Survival signaling induced by inflammatory cytokines or molecules	Selective ablation of tumor associated macrophages and blocking recruitment of tumor associated macrophages[124] Inhibition of JAKs and STATs [116] Non-steroidal anti-inflammatory drugs (NSAIDs) [116] Targeting cytokines such as IL-6 and TNF-α [116]

Genetic, epigenetic, and microenvironmental heterogeneity all contribute to therapy resistance mechanisms. Table 1 summarizes different strategies that have been employed to address these resistance mechanisms to varying degrees of success.

A significant limitation of current methods of tumor analysis at diagnosis is failure to capture the full spectrum of spatial and temporal tumor heterogeneity [3, 6], including the totality of genetic, epigenetic, or microenvironmental variations in a patient tumor. Consequently, targeted therapies selected based upon analysis of a small region of the patient tumor (current clinical practice) allows opportunity for Darwinian selection of cells that evade therapy [1, 3] and feed tumor recurrence. Tumor subtypes are currently identified by biomarker (such as HER2+ or HER2- breast cancers) or signature gene expression, or signaling pathway activity (such as Sonic Hedgehog (SHH) or Wnt pathways in medulloblastoma) within a small biopsy or region of a resected tumor. However, multiple spatial sampling of patient tumors has revealed that multiple genetically and epigenetically divergent subclones coexist in malignant tumors. For example, by sampling and analyzing four-six different regions of GBMs (Glioblastoma multiforme) from 11 patients, Sottoriva et a. reported significant heterogeneity in oncogenic driver mutations in different fragments of the same patient tumor [7]. In addition, gene expression analyses of different fragments also classified spatially separated fragments into different GBM molecular subgroups (proneural, neural, classical, or mesenchymal) [7], indicating that the molecular subtyping of an individual patient tumor is biased depending on which area of the tumor is analyzed. The area analyzed may represent only a portion of the original tumor if it was obtained during a biopsy, or it may be limited by the portion of a totally resected tumor that was used for analysis. In addition, single-cell RNA sequencing from five different GBM patient tumors showed that all five patient tumors consisted of individual cells that correspond to different molecular subgroups [8].

Extensive subclonal heterogeneity has also been described in many types of malignancy including but not limited to breast, pancreatic, colorectal, NSCLC, clear cell renal cell carcinoma, GBM, melanoma, acute lymphoblastic leukemia, and multiple myeloma [1, 3]. These observations clearly indicate that therapy selection based on single biopsies and/or determination of the molecular subtype based on the dominant expression signature from a mixed pool of cells from a selected region is unlikely to identify effective therapy that can eradicate all tumor cells. We anticipate that most patients with high-grade, malignant tumors will require multiple biopsy sampling and/or comprehensive molecular analysis of the majority of their resected tumors in order to identify optimal therapies. For successful treatment, combination therapies, either in parallel or in series, will often be necessary. Here, we review strategies for overcoming specific resistance mechanisms that operate at the genetic, epigenetic, and microenvironmental levels and discuss how these strategies can be combined to target multiple clones within a patient tumor.

## GENETIC HETEROGENEITY

Genetic heterogeneity in malignant cells arises from their increased tolerance for genomic instability, enabling them to successfully escape from cell death upon DNA damage, tolerate increased levels of chromosomal alterations and mutations, and acquire therapy-induced mutations [1, 3]. In some cases, severe genomic instability can involve chromothripsis or a hypermutator phenotype, in which cells with dysfunctional DNA repair machinery are selected for and many mutations are permitted to accumulate [1, 3]. As a result, neighboring cells in a given tumor mass can have different configurations of copy number changes, driver mutations, or passenger mutations [1, 3] (Figure 1). While many genetic aberrations are silent or non-functional, and these are not likely to play a major role in therapy response, significant intra-tumoral heterogeneity among known driver mutations, including HER2, EGFR, and PIK3CA has been reported [3]. Recent analyses of patient tumors provide compelling evidence for coexistence of genetically distinct subclones in a variety of tumor types [1, 3]. This observation has significant implications for prescribing specific therapies based on “molecular subtyping” of a small tumor sample, as currently practiced in the clinic, and indicate a need to change diagnostic practices to more comprehensively profile all oncogenic driver events in each patient tumor.

Paradoxically, pioneering studies have linked intermediate levels of genetic intra-tumoral heterogeneity with poor prognosis [9, 10],while extreme genetic heterogeneity has been associated with improved prognosis in some cases [9]. The underlying mechanism of any association between especially high genetic heterogeneity/mutational load and better clinical outcome remains unclear, though increased production of immunogenic neo-antigens and/or the deleterious effect of increased genetic instability have been proposed as potential mechanisms [9]. Nevertheless, moderate genetic heterogeneity, due either to the presence of pre-existing therapy-resistant clones or to emergence of treatment-induced mutations, appears to play a significant role in heterogeneous therapy response and resistance. For example, mutations in key survival pathways, such as the PI3K or p53 pathways, can confer cancer cells with different sensitivities to targeted therapy [11, 12], chemotherapy [13], and radiation [12], suggesting heterogeneity of cancer cells with these mutations could contribute to treatment resistance. In addition, genetic heterogeneity contributes to resistance to targeted therapies due to clonal selection of subclones that do not depend on the targeted pathway or to emergence of new mutations that bypass the targeted therapy. Due to space constraints, we summarize below only the approaches used to address different resistance mechanisms to targeted therapies resulting from genetically divergent clones in a tumor. We refer readers to this excellent review on the role of survival pathway mutations in resistance to cytotoxic therapy [13].

### Genetic heterogeneity and targeted therapy resistance

Targeted therapies affect a specific molecule or a group of molecules that share similar structure or function; hence, they are less toxic than cytotoxic chemotherapies and radiation therapy. Unfortunately, despite promising initial responses, many patients become resistant to targeted therapies. To date, two common mechanisms of resistance have been reported to targeted therapies.

**Figure 2:**
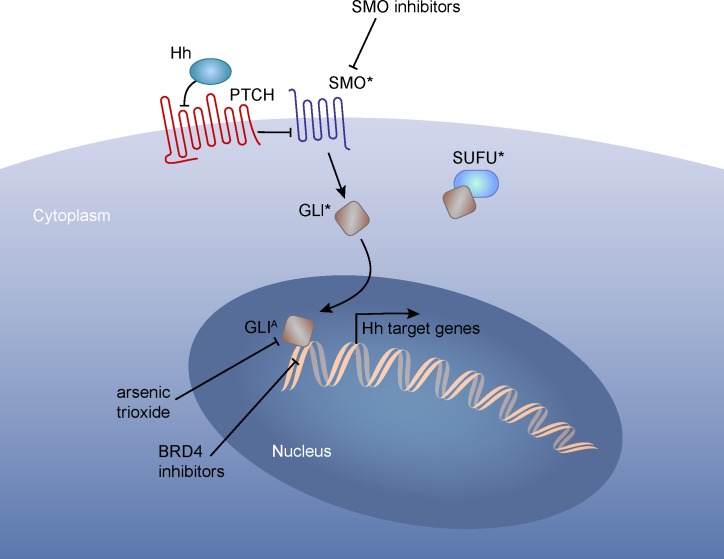
An example of different therapy resistance mechanisms to a targeted therapy Binding of SHH to its receptor, PTCH, results in release of SMO from PTCH inhibition. Therefore inactivation of mutations in Ptch or activating mutation of Smo results in elevated SHH signaling. Smoothen inhibitor-resistant SHH-driven tumors can acquire mutations at multiple levels: mutations within SMO, preventing drug binding; inactivating mutations in SUFU, a negative regulator of GLI nuclear localization; mutation or amplification of GLI transcription factors. Inhibitors that block GLI function, such as arsenic trioxide and BRD4 inhibitor (JQ1),has been shown to reduce proliferation of SMOi-resistant tumors (Tang et al., 2014) although new generation of BRD4 inhibitors may be necessary to prevent potential memory loss associated with JQ1 treatment (Korb et al., 2015). *:mutations, ^A^: amplifications.

The first common mechanism of resistance arises from secondary mutations in the drug target that prevent effective target inhibition by a drug. For example, secondary mutations in the BCR-ABL kinase domain that disrupt imatinib binding are common among resistant chronic myelogenous leukemia (CML) patients [14]. The resistance conferring T790M-EGFR mutation is common among NSCLC patients treated with EGFR tyrosine kinase inhibitors [15]. Mutations within the drug-binding pocket of the Smoothened (SMO) receptor are reported among basal cell carcinoma (BCC) and medulloblastoma patients who become resistant to the SHH/SMO inhibitor, Vismodegib [16, 17] (Figure 2).

The second common mechanism of resistance involves activation of “bypass” pathways. The bypass mechanism can involve the targeted pathway or a parallel pathway that can compensate for the targeted pathway. For example, alterations in upstream or downstream components of the targeted pathway can confer resistance to targeted therapy. It has been reported that melanoma treated with an oncogenic (V600E) BRAF inhibitor can become resistant by acquiring mutations in an upstream activator, N-RAS [18]. Also, Vismodegib- or NVP-LDE225- (SMO/SHH pathway inhibitors) resistant BCCs patients and mouse medulloblastoma cells have been shown to acquire mutations in *SUFU* or amplification of a downstream transcriptional effector of the SHH signaling pathway, *GLI2* [16, 19] (Figure 2). Oncogenic pathways may also be enhanced by de-repression of their suppressor pathways; inactivating mutation of the RAS antagonist NF1 confers resistance to BRAF inhibition in melanoma [20], and genetic lesions affecting PTEN (an antagonist of PI3K signaling) can confer resistance to inhibition of PI(3)Kα [21]. Bypass mutations also activate alternate pathways that can compensate for inhibition of the drug target. For example, genetic lesions that enhance PI3K signaling facilitate resistance to imatinib in CML [22], and activation of MAPK has also been implicated in resistance to SMO inhibitors in BCC patients and a mouse model of SHH medulloblastoma [23].

### Addressing mutation driven resistance mechanisms

Recent studies have demonstrated that, when acquired resistance emerges, targeting the same pathway using different drugs is a clinically viable approach. There are several examples of success with such an approach in the clinic. Second and third line inhibitors of BCR-ABL have shown success in CML patients resistant to imatinib [14]. Later generations of ALK inhibitors have been used to successfully treat NSCLC patients who have developed resistance to a first generation ALK inhibitor [24]. In addition, patients with the T790M EGFR mutation can be effectively treated with the third generation EGFR inhibitor Rociletinib after becoming resistant to other EGFR inhibitors [15]. Furthermore, there is some evidence that targeting the same molecule with multiple agents that work through different mechanisms can delay resistance. For instance, the combination of Trastuzumab and Pertuzumab, two agents that target HER2, along with docetaxel extends survival of HER2+ metastatic breast cancer patients compared to Trastuzumab and docetaxel alone [25]. As the number of FDA-approved targeted therapies increases, cycling through multiple generations of drugs that target the same critical oncogenic pathway may become an important strategy for extending survival.

Collectively, these observations suggest that it may be best to target oncoproteins from multiple angles. It is also clear that functional knowledge of the effects of different mutations on the same oncogene will provide tremendous value for optimizing personalized therapy. It should be noted, however, that the strategy to target the same oncoproteins using multiple targeted therapies might not be generalizable to all therapies. For instance, a variety of SMO mutants that are resistant to Vismodegib do not respond to other SMO inhibitors, (LDE225, LY2940680, and compound 5) [16]. In such cases, or when resistance results from activation of upstream or downstream components of the targeted pathway, targeting multiple nodes in the same pathway may be necessary. This concept has been tested in combinational therapies that target BRAF and MEK, core components of the MAPK pathway. Combining the BRAF inhibitor, dabrafenib, with the MEK inhibitor, trametinib, improves progression-free survival in melanoma patients with BRAF V600 mutations [26], and the same combination has demonstrated preliminary benefit in patients with metastatic NSCLC harboring the BRAF V600E mutation [27]. Combinations against multiple components of the same pathway could also prove superior in other cancer types. For example, tumors dependent on SHH signaling might be susceptible to co-targeting of SMO and the downstream GLI transcription factors (see Figure 2). Arsenic trioxide, which targets GLI2 for degradation, inhibits the growth of Vismodegib-resistant medulloblastoma *in vivo*, and the combination of arsenic trioxide and itraconazole, a SMO inhibitor, shows greater anti-tumor efficacy *in vivo* than either agent alone [28].

A significant rate-limiting step to targeting all clinically relevant subclones is comprehensive identification of all mutations or driver pathways present in an individual's tumor. Recent studies suggest that “liquid biopsy”, which utilizes analysis of tumor-derived, cell-free DNA (cfDNA) in the blood to detect mutations, may be useful in overcoming the spatial and temporal limitations of traditional biopsies [6]. cfDNA can identify mutations present in primary tumor tissue with a high specificity and sensitivity [29], and liquid biopsy has been used to detect mutations not detected in solid tumor biopsies [29, 30], suggesting that cfDNA can identify infrequent tumor subclones. cfDNA may also be used to monitor response to therapy; a decrease in mutant allele frequency in the plasma is often associated with disease stabilization or response, and an increase in mutant allele frequency can precede radiologic evidence of progression [31, 32]. Intriguingly, a variety of mutations that confer resistance to targeted therapies have also been detected in cfDNA, suggesting that liquid biopsy can be used to determine the mechanism of patient's resistance to treatment. Examples of these resistance associated mutations include KRAS, NRAS, MET, ERBB2, FLT3, EGFR and MAP2K1 for resistance to EGFR blockade in colorectal cancer [30], mutations in the estrogen receptor in breast cancer patients that progressed on aromatase inhibitors [33], alterations affecting the androgen receptor in prostate cancer patients who acquired resistance to anti-androgen therapy [34], and the T790M mutation in NSCLC patients that progressed on erlotinib [32].

Moving forward, cfDNA may be able to identify mutations that are clinically actionable. For example, in an NSCLC patient, cfDNA analysis identified a MET amplification that went undetected by 4 previous biopsies [35]. As more targeted agents are developed and treatment principles become more refined, resistance associated mutations identified in cfDNA might play a greater role in anticipatory treatments. Whether it is possible to diagnose and treat the cause of resistance by cfDNA alone remains to be seen. However, administration of agents which target the T790M mutation have already demonstrated some clinical benefit [15], and specific mutations in the estrogen receptor may suggest vulnerability to different endocrine therapies [33]. This implies that some of the resistance-conferring mutations that have been detected by cfDNA are already actionable. In addition, monitoring levels of resistance-granting mutations may even identify optimal dosing schedules. In a recent study, Siravegna et al. showed that K-RAS mutations associated with resistance to EGFR blockade were detectable in cfDNA with the emergence of resistance [30]. However, when EGFR blockade was discontinued, the frequency of resistance conferring K-RAS mutations in patient cfDNA declined [30]. Upon subsequent re-challenge with anti-EGFR antibodies, the tumor again responded to EGFR blockade, despite prior resistance [30]. These examples suggest liquid biopsy has clinical utility, although effective translation may require additional technological advances and validation, such as the need to develop standardized methodologies by which cfDNA is collected and analyzed [6]. For an in depth discussion of cfDNA technology and the obstacles which must be overcome to facilitate routine liquid biopsies, please refer to this excellent review [6].

A novel approach for overcoming genetic heterogeneity may involve immune checkpoint blockade therapy and immunotherapy more broadly. This is an active and rapidly growing area of research and merits its own expert review. Briefly, immune checkpoint blockade monoclonal antibodies stimulate an anti-tumor immune response by inhibiting activation of CTLA4 or PD-1, thus preventing cancer induced T cell anergy [36]. Cancer specific CD8+ T cells can then target tumor associated antigens and neoantigens to induce a therapeutic response [36, 37]. Immune checkpoint blockade has demonstrated clinical benefit in a variety of tumor types [36], particularly metastatic melanoma [38] and NSCLC [39]. A relatively consistent marker for improved clinical response to immune checkpoint blockade in melanoma and NSCLC is a higher tumor mutation and neoantigen load [40–44]. Immune checkpoint blockade is also more effective in colorectal cancer patients who have tumors that possess mismatch repair deficiency and a resulting high mutational load than in patients with mismatch repair proficient tumors and a lower mutational load [45].

Intuitively, a higher mutational and neoantigen load would indicate increased genetic heterogeneity, and therefore immune checkpoint blockade might be expected to provide strong therapeutic benefit against tumors containing a large number of genetic subclones. Nevertheless, mutational load does not necessarily correlate directly with the amount of genetic intra-tumoral heterogeneity. NSCLC patients who have a high neoantigen load still respond poorly when treated with immune checkpoint blockade if the majority of their neoantigens are subclonal, which reflects a high degree of intra-tumoral genetic heterogeneity [37]. Moreover, in a small sample of patients who responded well to immune checkpoint blockade, CD8+ T cells recognizing clonal neoantigens were detected but CD8+ T cells recognizing subclonal neoantigens were not, suggesting that immune checkpoint blockade may only stimulate an anti-tumor immune response against neoantigens present in all or most tumor cells [37]. Future work assessing immune checkpoint blockade in genetically heterogeneous tumors will help to clarify the relationship between therapeutic response, mutational load, and genetic intra-tumoral heterogeneity.

## EPIGENETIC HETEROGENEITY

In addition to genetic differences, cancer cells from the same tumor exist in different epigenetic states (Figure 1). Just as epigenetic changes accompany the lineage commitment and terminal differentiation that culminate in phenotypically divergent cell types among genetically identical cells during normal development, phenotypically different cancer cells expressing different RNAs and proteins co-exist among genetically identical cells within a tumor [3]. Mechanisms that sustain various epigenetic states are many and include DNA methylation, histone modifications, chromatin remodeling, and activities of non-coding RNAs [3, 46]. Intra-tumoral heterogeneity of DNA methylation patterns has been reported in human cancers [47, 48], and differences in gene expression and differentiation states of cancer cells have long been appreciated. Epigenetic modifications can be both heritable and reversible [3], which provides an especially fertile source of cancer-cell heterogeneity. Since the topic of this review is cancer cell heterogeneity and therapy resistance, we will focus our discussion on the role of cancer stem cells (CSCs) and epigenetic differences illustrated by stem *vs*. non-stem cell states. We will then discuss therapeutic strategies that might sensitize epigenetically resistant cancer cells to treatment.

### Cancer stem cells

CSCs, also referred to as “tumor initiating cells” or “tumor propagating cells”, provide a particularly apt illustration of the complexity that characterizes epigenetic heterogeneity. CSCs are defined as cancer cells with stem cell like-properties and tumor initiation capability. They sit at the apex of the cellular hierarchy, representing the most primitive or undifferentiated cell state [49]. The more differentiated progeny of CSCs are often referred to as “transit amplifying cells”, “bulk tumor cells”, or simply “non-CSCs” in different studies. Despite having stem cell characteristics, CSCs do not necessarily originate from normal tissue stem cells. Transformation of lineage committed progenitor cells can generate cancer cells that have the defining characteristics of CSCs: long-term self-renewal ability, regeneration of cellular hierarchy, and tumor initiation upon transplantation [50]. Various stem cell markers such as CD133 [51, 52], CD44 [53–55], and CD34 [56] have been used in different cancer types to enrich for cells showing the CSC phenotype. However, these are not universal markers for CSCs, even within the same clinical tumor type. For example, while CD133 expression was used to identify glioma stem cells in early studies, CD133 negative cancer cells have also been reported to display the CSC phenotype in subsequent studies [52, 57]. Because of the lack of a definitive cell surface marker for CSCs, other studies have taken advantage of unique cellular characteristics of stem cells, such as high aldehyde dehydrogenase (ALDH) or ATP binding cassette (ABC) transporter activity to identify CSCs in ALDH+ or side-population (SP) cells, respectively [50, 58]. However, to the best of our knowledge, no marker or assay for CSCs with perfect positive- or negative- predictive value has yet been identified for any tumor type. This lack of definitive markers is a challenge that is likely to persist as it reflects inter- and intra-tumoral heterogeneity of human cancers.

Relevant for this review, it has been shown that CSCs survive current treatments and fuel tumor recurrence. In addition to studies reporting enrichment of CSCs after treatment in patients [59, 60], Parada and colleagues used a mouse model of glioma to experimentally show that cells expressing the neural stem cell marker Nestin survive temozolomide and radiation treatment and seed recurring tumors *in vivo* [61]. CSCs have been shown to be more resistant to chemotherapies, radiation, and targeted therapies than bulk tumor cells through multiple mechanisms. First, similar to normal stem cells in adult tissues, CSCs in some tumors have been shown to be quiescent [50]; therefore, therapies that target fast dividing cells (most chemotherapies) are ineffective in these cells. Second, again similar to normal stem cells, CSCs express high levels of ABC transporters that efflux harmful chemicals, including chemotherapies [50]. In fact, the side-population phenotype arises from the ability of ABC transporters to extrude Hoechst 33342 dye in normal and cancer stem cells [62]. Third, CSCs have enhanced ability to repair DNA damage and survive ionizing radiation [63, 64]. Fourth, CSCs and bulk tumor cells may depend on different mitogenic and survival pathways. We recently showed that CSCs retain epigenetic memory of their cells of origin, including mitogenic signaling pathways, that can differ from those that drive bulk tumor cell proliferation and survival. Therefore, the targeted therapy selected to block mitogenic signaling in bulk tumor cells was ineffective in suppressing CSC proliferation/survival [65]. Fifth, CSCs express higher levels of anti-apoptotic and pro-survival genes than bulk tumor cells, leading to an enhanced ability to survive treatment [66–69].

The CSC compartment within a tumor may also be heterogeneous and include CSCs that possess distinct transcriptional signatures and marker expression patterns [70–72]. For example, we showed that in the *Ptch*+/− mouse model of medulloblastoma, CSCs with different molecular phenotypes arise from different cells of origin during brain development [65]. Importantly, while at the bulk tumor level, all tumors are classified as the SHH tumor subtype based on strong SHH pathway gene expression, CSCs derived from transformed neural stem cells did not depend on SHH signaling for proliferation or survival. In other words, in a subgroup of SHH medulloblastoma, the bulk tumor cells and CSCs depend on different mitogenic and survival signaling pathways [65]. We also showed that Ptch medulloblastoma CSCs can evolve *in vivo* and *in vitro* [65], suggesting that CSC heterogeneity can affect tumor phenotype in a clinically relevant manner.

Finally, CSC heterogeneity is likely to contribute to tumor recurrence and act as a vital mechanism by which tumor heterogeneity persists despite treatment that eliminates the non-CSC population in tumors. For example, Sharma et al. studied the therapy response of multiple human cancer cell lines to anti-cancer agents and observed that a small fraction of reversibly drug-tolerant cells existed in each cell line [73]. In addition to being reversible, the drug-resistant cellular state depended on the histone demethylase KDM5A and IGF-1 signaling [73], highlighting an epigenetic mechanism of therapy resistance. Similarly, a longitudinal study involving 47 breast cancer patients undergoing neoadjuvant therapy showed that pre- and post-treatment tumors do not show significant shifts in genetic heterogeneity but do show strong phenotypic/epigenetic changes post treatment, marked by reduced numbers of cells exhibiting a differentiated phenotype (CD24+/CD44−) and increased numbers of cells displaying a mesenchymal or stem like phenotype (CD24−/CD44+) [60]. These *in vitro* and *in vivo* studies suggest that combination therapies including inhibitors of epigenetic regulators may enhance the efficacy of chemo- and targeted therapies.

In addition to multiple cellular mechanisms that protect CSCs from conventional and targeted therapies, studies have shown that bulk tumor cells can acquire CSC phenotypes spontaneously [74] and in response to microenvironmental signals such as TGFbeta, hypoxia, and acidosis [74–77]. In other words, cellular hierarchy in malignant tumors can be bidirectional and dynamic, with non-CSCs being able to “reverse” into a CSC state. For example, Chaffer et al. showed that transformed human mammary epithelial cells with a non-CSC CD44 low phenotype spontaneously adopt a CSC CD44 high phenotype both *in vitro* and *in vivo* [74]. One well-studied mechanism by which non-CSCs may revert to a CSC phenotype is the epithelial-mesenchymal transition (EMT), as cells that have undergone EMT can express CSC markers and demonstrate an increased ability to initiate tumors [78]. For example, Mani et al. found that ectopic expression of the EMT inducing transcription factors *Snail* or *Twist* in non-tumorigenic, immortalized human mammary epithelial cells induced the breast CSC CD44^high^/CD24^low^expression profile [78]. EMT induction also enhanced the ability to form mammospheres *in vitro* and induce tumors *in vivo* [78]. The EMT inducing transcription factor ZEB1 also facilitates conversion of CD44^low^ breast cancer cells into a CD44^hi^ CSC state [75]. Moreover, shRNA knockdown of ZEB1 in breast cancer cells impairs tumor initiation ability, suggesting that conversion of non-CSCs into a CSC state is important for tumorigenesis [75]. Likewise, in many tumors a “mesenchymal” phenotype is associated with therapy resistance, consistent with its association with the CSC state [50, 66]. Consistently, CSCs and EMT are regulated by overlapping signaling pathways and transcription factors, and can be induced by similar microenvironmental stimuli [66, 79]. In addition, powerful stem cell regulators including YAP [80–82] and c-MYC [83, 84] can mediate treatment-resistant epigenetic states, and WWTR1/TAZ has been shown to promote the CSC phenotype and EMT in breast tumors [85]. These observations suggest the CSC state may be achieved through multiple mechanisms, and an effective treatment strategy for targeting CSCs would also require blocking regeneration of CSCs from bulk tumor cells.

### Targeting epigenetically driven therapy resistance

There are two broad strategies for sensitizing cancer cells in epigenetically resistant states to treatment. First, it is possible to directly target resistance mechanisms that are sustained by epigenetic modifications and allow cancer cells to survive therapy. Table I lists many therapies that target specific mechanisms of resistance, such as heat shock proteins or drug efflux pumps, that are often expressed by cancer cells in resistant epigenetic states. In addition, it may be possible to shift CSCs [86], cells that have undergone EMT [87], and other therapy resistant cancer cell states [88] into an epigenetic state that is more vulnerable to treatment; e.g., through “differentiation therapy”.

The second approach is to manipulate the epigenetic machinery using inhibitors of the enzymes that epigenetically modify DNA and histones. Although many inhibitors of these enzymes have been described, we will limit our discussion to DNA methyltransferase inhibitors (DMTis) and histone deacetylase inhibitors (HDACis), for which significant amount of clinical experience exists. Widely studied DMTis include decitabine and 5-azacitidine, and well-investigated HDACis include romidepsin and vorinostat. Decitabine and 5-azacitidine are FDA-approved for myelodysplasia and can be useful for treating acute myeloid leukemia (AML), while romidepsin is approved for cutaneous T cell lymphomas and for relapsed peripheral T cell lymphoma [89].

In a variety of pre-clinical models of both solid tumors and hematological malignancies, DMTis and HDACis demonstrate significant anti-tumor efficacy and sensitize many cancer types to chemotherapy [89, 90], radiation [91], and immune checkpoint blockade [92]. They accomplish this *via* multiple mechanisms. First, they can activate expression of tumor suppressor genes, apoptosis-related genes, and pro-differentiation genes, rendering a cancer cell more vulnerable to therapy [89, 90]. DNA methyltransferases methylate promoters and other regulatory regions in the genome to silence transcription from nearby genes, and histone deacetylases remove acetyl groups from histones, converting chromatin regions to inactive states [89]. Therefore, DMTi and HDACi treatments can induce expression of tumor suppressor genes that were silenced in cancer cells by DNA methylation or histone deacetylation [89]. Second, DMTis and HDAC can be cytotoxic. HDACis can enhance cancer cell vulnerability to genotoxic chemotherapy by creating an open chromatin state that is more vulnerable to genotoxic stressors [93], and most DMTis incorporate directly into DNA to cause DNA damage, particularly when used at high doses [46]. Third, DMTis and HDACis may induce differentiation of CSCs or change their epigenetic states such that they are more vulnerable to treatment. For example, a novel DMTi, SGI-110, sensitizes ovarian CSCs to platinum therapy and induces expression of differentiation-associated genes [86]. SGI-110 also enhances cisplatin-induced DNA damage and demonstrates anti-tumor efficacy in an ovarian cancer xenograft model [90]. Furthermore, treatment with DMTis reduces the percentage of cells showing CSC markers in breast cancer cell lines [94], and HDACi treatment eliminates NSCLC cells resistant to both EGFR tyrosine kinase inhibitors and cisplatin [73]. Fourth, epigenetic drugs may also be able to suppress EMT. The HDACi, mocetinostat, sensitizes pancreatic cancer xenografts to gemcitabine in association with upregulation of E-cadherin and downregulation of the EMT inducing transcription factor ZEB1 [87]. Together, these studies suggest potential therapeutic effects of DMTis and HDACis through multiple mechanisms.

Unfortunately, initial clinical trials with DMTis and HDACis in solid tumors were not successful. This was mainly due to relatively high doses of drugs used in those trials, designed to induce a cytotoxic effect, which resulted in unacceptable levels of toxicity and precluded definitive analysis of efficacy [89]. More recent clinical trials using reduced dosages demonstrated anti-cancer activities of DMTis and HDACis in hematological malignancies [89]. In contrast, epigenetic therapies as single agents have not demonstrated significant clinical efficacy in solid tumors yet. Only a few clinical trials to evaluate the efficacy of combination therapy incorporating DMTis and HDACis have been reported at the present [89]. Low dosages of the DMTi decitabine, administered before carboplatin treatment, can re-sensitize heavily pretreated and resistant ovarian cancer patients to platinum therapy [95]. In lung cancer, combined epigenetic therapy using a DMTi and an HDACi (azacitidine and entinostat, respectively) showed modest benefit for treating metastatic NSCLC [96], and a HDACi, vorinostat, has been shown to enhance the efficacy of carboplatin and paclitaxel in patients with previously untreated advanced NSCLC [97].

While the above results suggest potential efficacy for some epigenetic modulator inhibitors in treating solid tumors, their routine use in the clinic may be premature. Not all clinical trials have demonstrated the ability of these drugs to enhance the effectiveness of other treatments. For example, Entinostat did not improve the efficacy of erlotinib in patients with stage IIIB/IV NSCLC, despite supportive pre-clinical evidence [98]. A major concern is that epigenetic modulators are not specific and that it is not possible to select the regions of the genome that are affected by DMTi or HDACi treatments. In some contexts, DMTis and HDACis might activate genes that make cancer cells more resistant to therapy. For example, previous studies have shown that HDACis can stimulate expression of drug efflux pumps in cancer cell lines and cause subsequent multi-drug resistance, though it is worth noting that other studies have reported that HDACis can suppress expression of drug efflux pumps [99]. This underlines the unsettling proposition that epigenome-modifying drugs could function as double-edged swords: they could induce anti-tumor and therapy sensitizing effects in some contexts but cause tumor progression and treatment resistance in others. Consequently, perhaps DMTi and HDACi treatments should be reserved as a “last-ditch effort” against heavily pre-treated and therapy-resistant tumors (that presumably acquired drug-tolerant epigenetic states) and not used in patients who are sensitive to other therapies. In addition, reversibility of the cancer cell hierarchy implies that the effect of epigenetic modulator inhibition may be transient. For instance, the bidirectionality of the CSC hierarchy, in which non-CSCs can acquire CSC phenotypes [74, 75], suggests that non-CSCs might be able to dedifferentiate and reconstitute the CSC population in response to a CSC targeting therapy. Moving forward, it will be necessary to investigate the influence of starting genetic and epigenetic states on response to epigenetic modifying drugs, identify biomarkers to identify patients who will benefit from DMTi and HDACi treatments, and determine the safe order and scheduling of combination therapies that include epigenome-modifying drugs.

## MICROENVIRONMENTAL HETEROGENEITY

Cancer cells reside in microenvironments that can be drastically different (Figure 1), which adds another layer of complexity and heterogeneous therapy response even among genetically identical cells. Tumor tissues contain gradients of growth factors, cytokines, oxygen, and nutrients, as well as differences in extra-cellular matrix (ECM), pH, and vascularization [5, 76]. In addition, proximity to stromal cells such as immune cells and fibroblasts, which can function in paracrine manners to support cancer cell proliferation and survival [4, 5], also differs among cancer cells. This extensive variation in the tumor landscape creates numerous microenvironmental niches (Figure 1), and genetically identical cancer cells can be differentially sensitive to the same treatment, depending on the particular microenvironmental niche in which they reside [3, 4]. In this section, we review the role of tumor microenvironment in treatment response and tumor heterogeneity, and we discuss strategies to target cancer cells in resistant microenvironmental niches or alter the tumor microenvironment to sensitize cancer cells to therapy. Since tumor microenvironment is a fertile research area, it is impossible to review it comprehensively in a limited space. We will limit our discussion to two well-described microenvironmental factors that contribute to tumor cell heterogeneity and therapy response: biophysical properties and inflammation.

### Heterogeneity in biophysical properties

Different geographical positions within a tumor are composed of varying landscapes with different pockets of biophysical properties such as vascularity, hypoxia, pH, and ECM organization [5, 76]. A major contributor to such microenvironmental heterogeneity is poor and aberrant vascularization within malignant tumors, which provides the tumor mass with uneven access to oxygen, nutrients, blood-borne drugs, and immunological effectors [100]. Poor vascularization can prevent a sufficient diffusion of systemically administered therapy from reaching all tumor cells at an effective concentration [100]. In addition, leaky tumor vessels create increased interstitial fluid pressure in tumors relative to the surrounding healthy tissue, further reducing drug diffusion [100]. Moreover, lack of blood flow to a tumor can also impair the cytotoxic effects of some treatments, such as radiation, which depends on tissue oxygenation, and of immunotherapy, which requires immune-cell infiltration [100]. These observations have led to a variety of approaches to normalize the aberrant tumor vasculature (Table 1). In particular, smaller doses of angiogenesis inhibitors have been used to normalize the tumor vasculature and increase therapeutic benefit by improving tumor perfusion [100–102]. The benefits of commonly prescribed inhibitors of angiogenesis such as Avastin/bevacizumab have been modest, but it has been proposed that this may be due to inappropriate dosing and that lower doses could provide greater therapeutic benefit [100]. For an in-depth discussion of this hypothesis and other clinical considerations regarding the use of anti-angiogenic therapy as a vasculature normalization agent, please see this expert review [100].

Many anti-cancer therapies currently used in the clinic are toxic because they generate high levels of reactive oxygen species, which requires oxygen; therefore, cells in hypoxic environments are thought to be resistant to such therapies [103]. Hypoxia arises in rapidly growing tumors that fail to recruit sufficient blood vessels. The mechanisms by which tumor hypoxia may create a drug-resistant state are numerous and diverse. A major mechanism through which hypoxia affects cancer cell behavior is induction of the transcription factor HIF-1α (hypoxia induced factor-1α). Hypoxia and HIF-1α can increase expression of drug efflux pumps, induce cell cycle arrest in G1 or G2, and alter metabolism to facilitate cancer cell survival [103]. HIF-1α also promotes processes vital for tumor progression including angiogenesis, enhanced utilization of glucose, and metastasis [103]. Furthermore, hypoxia can also induce autophagy, which can promote survival and therapy resistance in some contexts [104]. In addition, evidence suggests that hypoxia also poses a challenge to immunotherapy; CD8+ T cells and CD4+ T cells avoid hypoxic areas of tumors and demonstrate an immunosuppressed phenotype when present [105]. Moreover, hypoxia can select for cancer cells with inherent genetic and epigenetic resistance mechanisms by decreasing expression of pro-apoptotic proteins and selecting for TP53 mutant cells that are resistant to apoptosis [103]. Finally, hypoxia can also promote EMT and CSC phenotypes [103].

Despite the many mechanisms through which hypoxia can induce resistance, there are multiple strategies to target hypoxic cancer cells (Table 1). Space limitations permit discussion of only a few of them. Hypoxia can arrest cells in S-phase, which sensitizes them to PARP-1 inhibition [103]. Some hypoxic cancer cells are also defective in nucleotide excision repair and homologous recombination dependent repair, which can increase susceptibility to DNA cross-linking agents such as cisplatin [103]. Although these vulnerabilities must be weighed against the challenges of drug delivery and drug efflux pump expression, it is possible that judicious selection of established therapies might more effectively target cancer cells in hypoxic microenvironments. Hypoxic cancer cells can also be eliminated or sensitized to treatment by inhibition of HIF-1α-dependent transcription through agents such as the cardiac glycoside digoxin, acriflavine, and the small molecule HIF-1α inhibitor PX-478 [106, 107].

Acidosis is another physiological property that can mediate therapy resistance. Acidosis is a commonly observed phenomenon in malignant solid tumors, presumably arising from poor vascularization, resulting in hypoxia, reduced cellular waste elimination, and increased glycolysis [76, 103]. Particularly relevant to therapy resistance, acidosis can induce a CSC phenotype in GBM cells, including increased self-renewal and tumor initiation ability [76]. In addition, reversed gradients of intra- and inter-cellular pH in acidic microenvironments can have a significant impact on the cellular uptake of anti-cancer agents that are pH-sensitive [103].

Finally, diffusion of cancer therapies may also be impeded by the stiff, collagenous, and dense ECM (i.e.,desmoplastic stroma) that is common to many solid tumors [100]. Biomechanical forces from the desmoplastic stroma can also affect gene expression and therapeutic response. For example, stiffness activates the Hippo/YAP/TAZ pathway, which then provides HER2 amplified breast cancer cells with resistance to lapatinib [108]. The Hippo/TAZ pathway also maintains the breast CSC phenotype [85] and chemoresistance [109]. Cancer cell-ECM interactions can also maintain the CSC niche [110] and provide cancer cell resistance to chemotherapy, radiation and targeted agents [110, 111]. For instance, FAK and Src kinases are believed to be vital to integrin activation of pro-survival pathways, and subsequent induction of anti-apoptotic proteins can confer apoptosis resistance [110]. As such, inhibitors of FAK, Src, anti-apoptotic proteins, and specific integrins can sensitize matrix-attached cancer cells to treatment in pre-clinical models (Table 1).

Altering the ECM through ECM modulators might have the potential to increase the penetrance of chemotherapy into solid tumor tissues and thereby enhance treatment efficacy. Stiff collagenous ECM impedes the diffusion of macromolecules into tumors, and degradation of collagen using bacterial collagenase can dramatically improve permeability [100]. Bacterial collagenase is not ideal for systemic administration, but the commonly prescribed angiotensin II receptor blocker (ARB) losartan can increase tumor permeability by inhibiting collagen production [100]. In mouse models, losartan increases the efficacy of liposomal doxorubicin against desmoplastic tumors [112]. Other methods of altering the ECM to increase permeability of cancer therapy are described in Table 1. However, caution may be warranted before targeting the desmoplastic stroma routinely, as a dense stroma has been shown to constrain tumor progression in some contexts [113]. Likewise, matrix metalloproteinases (MMPs), which degrade and remodel ECM, can be potent facilitators of tumorigenesis and progression [114]. For example, MMP3/stromelysin-1 promotes mammary tumorigenesis in mouse models [115]. The role of ECM degradative MMPs in tumorigenesis and progression suggests that targeting the desmoplastic stroma will require a sophisticated understanding and focus on stromal remodeling rather than destruction. MMP inhibitors have failed to demonstrate anti-cancer efficacy in clinical trials as of yet [114].

### Heterogeneity of stromal cells and inflammatory factors

While past treatment efforts have mainly focused on eradicating transformed cancer cells, recent studies highlight the importance of non-tumor cells, or tumor stroma, and their roles in tumor progression and therapy response. Major constituents of tumor stroma include cancer-associated fibroblasts (CAFs) and immune cells [4, 5]. The infiltrating immune-cell population includes myeloid-derived suppressor cells [116], tumor-associated macrophages (TAM) and neutrophils [116], T lymphocytes [116], and additional cell types. Stromal cells secrete various factors that can affect the epigenetic states of cancer cells and modulate therapy response and cancer cell survival.

Inflammatory cytokines and other factors derived from infiltrating immune cells and other stromal components may induce survival signaling in cancer cells that facilitates resistance to treatment. Among others, STAT3 and NF-κB are major signaling pathways downstream of inflammatory stimuli, which can facilitate resistance to chemotherapy, radiation, and targeted agents [117–122]. Important activators of STAT-3 include IL-6 and IL-11, which are often over-expressed in cancers [123], and inducers of NF-κB transcription include toll like receptor ligands, tumor necrosis factor alpha (TNF-α), and interleukin-1β [120]. NF-κB and STAT3 activation and crosstalk are complex, as activation of each transcription factor may be induced by a partially overlapping set of cytokines, extracellular factors, and signaling pathways [116, 120, 123]. Moreover, NF-κB can induce expression of a variety of molecules, including interleukin-6 (IL-6), that activate STAT3 [123]; some genes may be induced by both NF-κB and STAT3; and STAT3 can enhance and prolong NF-κB mediated transcription [120].

Agents that modulate tumor-associated immune cells, including TAMs, or target individual cytokines, includingIL-6, have had some success in pre-clinical models [123, 124]. FDA-approved anti-TNF-α and anti-IL-6 therapeutics have been successfully introduced to treat chronic inflammatory diseases, but as of yet little clinical efficacy has been observed in solid tumors [116, 125]. There are multiple potential explanations for this observation including functional redundancies among various inflammatory cytokines. Therefore, agents that can prevent STAT3 and NF-κB mediated transcription and therefore act more downstream than inhibitors of specific cytokines are desperately needed. We are unaware of any NF-κB-specific targeting agents that are clinically available, but many STAT3 inhibitors are currently in development, as are inhibitors of JAKs, which are vital STAT signaling partners [116]. JAK inhibition has proved useful in hematological malignancies [116], and clinical trials of STAT3 and JAK inhibitors in solid tumors are ongoing. However, the road to clinical development for these agents may be long and arduous; to the best of our knowledge neither STAT3 nor JAK inhibition has yet demonstrated efficacy against solid tumors in published clinical trials [126, 127].

### Additional secreted factors

Cancer cells and components of the tumor stroma, including CAFs and TAMs, secrete a variety of growth factors and pro-survival molecules that act through paracrine mechanisms to provide resistance to treatment [4]. For example, Wilson et al. exposed 41 oncogene addicted cancer cell lines to a panel of six growth factors that included hepatocyte growth factor (HGF), epidermal growth factor (EGF), fibroblast growth factor (FGF), platelet-derived growth factor (PDGF), neuregulin 1 (NRG1) and insulin-like growth factor (IGF) [128]. Most of the cell lines had distinct oncogenic kinase dependencies and displayed sensitivities to relevant RTK inhibitors; however, when one or more of the exogenous growth factors were added, they became less responsive to the original drug [128]. Rescue was achieved *via* activation of PI3K-AKT or MAPK through signaling of secondary RTKs rather than the RTK targeted by the drug [128]. For example, HGF signaling through its receptor (MET) facilitated resistance to lapatinib in HER2 amplified breast cancer cells *via* activation of PI3K and MAPK [128]. Although it may be difficult to confirm that endogenous growth factors can compensate for RTK inhibition in the therapeutic context, this study suggests that it may be prudent to utilize more downstream inhibitors of PI3K or MAPK in combination with agents that act on upstream kinases like HER2 or EGFR.

Intriguingly, one additional paracrine source of resistance may be exosomes [129, 130], which are lipid bound “cargo containers” that can transfer proteins, DNA, and RNA from one cell to another [129]. For example, it has been reported that CAFs secreting exosomes that contain the Wnt ligand, Wnt3A, increase the resistance of colon CSCs to chemotherapy [130]. Exosomes can also grant chemoresistance by transferring anti-apoptotic microRNAs or drug efflux proteins from resistant cancer cells to their neighbors [129]. Therefore, pharmacological inhibition of exosome secretion might help sensitize cancer cells to therapy [129, 130].

## PERSPECTIVE

### Towards personalized combination therapies that account for tumor heterogeneity

The future of oncology will likely require designing individualized treatment combinations that consider intra-tumoral heterogeneity at genetic, epigenetic, and microenvironmental levels and address the therapy resistance mechanisms (anticipatory therapy [131]) that operate in a tumor. Even if clinical practices change such that each patient tumor can be comprehensively scrutinized, there are several significant hurdles that must be overcome to achieve effective personalized care. The first is the establishment of a vast armory of cancer therapies that can be “picked off the shelf” when an individual needs them. The vast majority of agents described in Table 1 have not yet achieved FDA approval, and more novel therapies are desperately needed. Going forward, clinical trials must utilize appropriate biomarkers to select suitable patient pools to measure the efficacy of new therapies. It may be prudent to consider the total spread of patient responses to novel therapeutics, rather than mean or median response, as this will enable development of agents that might be effective in limited circumstances to be used in appropriate but rare cases. Building a vast arsenal of safe and well-tolerated cancer drugs in this way would establish a powerful substrate from which patient-specific combinations could be designed.

The second major challenge is designing and implementing mini-clinical trials for testing new combinations of therapies in a safe, efficient, and cost-effective way. As the number of identified driver oncogenes/pathways grows by increasing numbers of large-scale cancer genomics analyses, and as the number of available targeted therapies increases, there will be an exponential increase in permutations of potential combinations that can be used to individualize patient treatment. However, the number of patients expected to benefit from a given treatment regimen will decrease the more “personalized” or specific the treatment combinations become. This reality will require a new clinical trial paradigm that allows safety and efficacy testing of novel combinations in small numbers of properly selected patients. An interesting alternative may be the use of patient derived xenografts (PDX) models and/or mouse avatars. For example, Novartis generated a panel of over 1,000 PDX models containing a variety of tumor types and driver mutations [132]. They used these models to perform a large-scale 1×1×1 (1 mouse representing 1 patient tumor in1 treatment group) trial to retrospectively verify the results of clinical trials testing BRAF inhibitors and the BRAF/MEK inhibitor combination in BRAF mutant melanoma [132]. While this minimalistic approach was not highly predictive of individualized response, the population level analysis correlated well with patient population response [132]. Likewise, mouse avatars, which are PDX models derived from patients currently undergoing treatment, have shown some success in selecting clinically beneficial therapies for individual patients [133]. Together, these studies suggest judicious use of PDX and avatar models can facilitate testing of new therapy combinations in a safe, efficient, and cost-effective way.

The third challenge in customizing combination therapy is the size and complexity of the datasets that should be considered for personalized therapy. Ideally, combinations should be derived from integrating the genetic, epigenetic, and microenvironmental heterogeneity data, including mechanisms of pre-existing resistance at the start of treatment. In addition, biomarkers for anticipated resistance mechanisms should be assessed periodically to detect the emergence of resistant clones. In the future, combinations maybe rationally designed, implemented, and adjusted to include agents that eliminate stubborn clones or sensitize resistant cancer cells to therapy. Combination design might also consider tumor subtype, the sequence and scheduling of therapies, markers of minimal residual disease, and patient genomic data. In short, future design of combination therapies will be extremely complicated and will probably rely on “big data” approaches that consider myriad factors.

A related challenge is the necessity of developing better models of intra-tumoral heterogeneity. An emerging technology that may improve our ability to model genetic intra-tumoral heterogeneity is the clustered regularly interspaced short palindromic repeats (CRISPR) /CRISPR associated protein 9 (Cas9) system. Unlike traditional transgenic methods, CRISPR/CAS9 can inactivate multiple tumor suppressor genes or introduce activating mutations in oncogenes simultaneously, either in cell culture or in mice *in vivo* [134]. Moreover, sequencing of multiple regions of the same tumor can also infer which of the induced driver mutations tend to occupy a trunk position on the phylogenetic tree of the tumor and which tend to be less selected for and remain subclonal [134]. By combining genomic information with CRISPR/CAS9 system, faithful models that reflect tumor evolution can be use to evaluate therapy responses of different subclones.

In the near future, the anti-cancer arsenal will include cytotoxic chemotherapies, radiation, targeted therapies, and new classes of drugs that modulate cancer cell metabolism and the immune system. Cytotoxic chemotherapy and radiation will likely remain staples of cancer treatment for some time since it may be difficult to develop targeted therapies for some oncogenic drivers and acquired resistance to targeted therapies is common occurrence. Targeted therapies will likely become more effective as we develop the ability to target multiple pathways (MAPK, PI3K-AKT-mTOR, Hedgehog, etc.) and multiple effectors in the same pathway (i.e., BRAF and MEK inhibition). Furthermore, targeting downstream pathway components, such as JAKs, STAT3, AKT, or MEK, may be preferable to targeting individual upstream signaling activators such as growth factors, cytokines, and receptors, as it reduces the number of potential bypass points. Epigenetic therapy might be effective in highly treated patients where most cells are in a drug-resistant epigenetic state. It will be important to develop treatments that can modulate the tumor microenvironment, such as hypoxic and acidic niches, as these can induce drug-resistant cell states through a variety of mechanisms. Finally, targeting crucial pro-survival factors, including BCL-2 family members, DNA repair enzymes, and heat shock proteins might be vital to the combinations of the future, though caution is warranted as inhibiting important survival molecules might sensitize normal cells to cytotoxic treatment and produce unacceptable toxicity.
